# Mouse model of chromosome mosaicism reveals lineage-specific depletion of aneuploid cells and normal developmental potential

**DOI:** 10.1038/ncomms11165

**Published:** 2016-03-29

**Authors:** Helen Bolton, Sarah J. L. Graham, Niels Van der Aa, Parveen Kumar, Koen Theunis, Elia Fernandez Gallardo, Thierry Voet, Magdalena Zernicka-Goetz

**Affiliations:** 1 Department of Physiology, Development and Neuroscience and Gurdon Institute, University of Cambridge, Downing Street, Cambridge CB2 3EG, UK; 2 Department of Human Genetics, University of Leuven, KU Leuven, Leuven 3000, Belgium; 3 Sanger Institute-EBI Single-Cell Genomics Centre, Wellcome Trust Sanger Institute, Hinxton CB10 1SA, UK

## Abstract

Most human pre-implantation embryos are mosaics of euploid and aneuploid cells. To determine the fate of aneuploid cells and the developmental potential of mosaic embryos, here we generate a mouse model of chromosome mosaicism. By treating embryos with a spindle assembly checkpoint inhibitor during the four- to eight-cell division, we efficiently generate aneuploid cells, resulting in embryo death during peri-implantation development. Live-embryo imaging and single-cell tracking in chimeric embryos, containing aneuploid and euploid cells, reveal that the fate of aneuploid cells depends on lineage: aneuploid cells in the fetal lineage are eliminated by apoptosis, whereas those in the placental lineage show severe proliferative defects. Overall, the proportion of aneuploid cells is progressively depleted from the blastocyst stage onwards. Finally, we show that mosaic embryos have full developmental potential, provided they contain sufficient euploid cells, a finding of significance for the assessment of embryo vitality in the clinic.

## Abstract (web summary)

The developmental potential of mosaic embryos of euploid and aneuploid cells is unknown. Here, the authors create a mouse model of chromosome mosaicism, showing that aneuploid cells in the fetus are eliminated by apoptosis and developmental potential is dependent on the presence of sufficient euploid cells.

## Graphical abstract

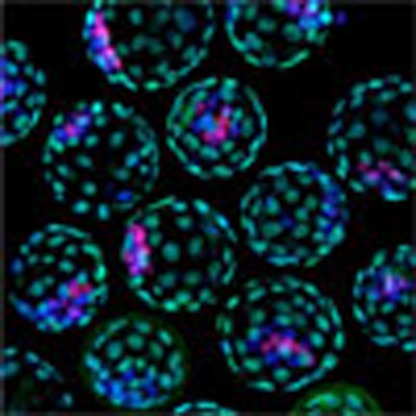

The majority of human pre-implantation embryos display chromosome mosaicism, with the most common pattern being euploid–aneuploid mosaicism where the embryo contains a complement of both normal and abnormal cells^1^. This mosaicism arises due to an error in mitosis during the first few cleavage divisions following fertilization and is believed to be directly responsible for the high rates of early human pregnancy failure in both spontaneous conceptions^2^ and following *in vitro* fertilization (IVF)^3^,^4^,^5^,^6^. Despite the high incidence of mosaicism in human pre-implantation embryos, the fate of aneuploid cells in the embryo is not clear and many studies in human embryos rely on morphological features to assess embryo development. Chromosome mosaicism is most frequently observed in embryos at the early cleavage stages, declining in prevalence as gestation progresses^1^,^7^. Whether this shift results from developmental failure of the whole embryo or alternatively through elimination of abnormal cells remains currently unknown. Observational findings comparing mosaicism levels with IVF outcomes suggest that some mosaic embryos can develop into viable pregnancies^8^,^9^. If indeed some mosaic embryos have full developmental potential, it is important to understand what confers their viability.

By using a mouse model for chromosome mosaicism, it is possible to use methodological strategies that are not possible in human embryos. At the morphological level, mouse pre-implantation development is similar to that in humans, undergoing cleavage divisions, compaction, blastocyst cavity formation and hatching, albeit with slightly different timings^10^,^11^,^12^. Both mouse and human pre-implantation development culminates in the formation of a blastocyst that is composed of the extra-embryonic trophectoderm (TE) and primitive endoderm (PE), which will form the placenta and yolk sac, respectively, and the embryonic epiblast (EPI), which forms the fetus^12^,^13^. These cell lineages are specified in two cell fate decisions. In the first cell fate decision, cells on the outside of the embryo form the TE, whereas cells on the inside form the pluripotent inner cell mass (ICM). In the second cell fate decision, cells of the ICM are segregated into the PE and the EPI. The correct specification of these lineages and the formation of a blastocyst able to implant are essential for all subsequent development^13^. Here we have generated a mouse model of pre-implantation chromosome mosaicism and have investigated both the developmental fate of aneuploid cells and the consequences of mosaic aneuploidy for successful development of the whole embryo. By determining the development of mosaic embryos at single-cell resolution, we show that aneuploid cells become eliminated from the embryo, starting just before implantation, and that mosaic euploid–aneuploid embryos have comparable developmental potential to normal embryos, provided they contain a sufficient proportion of euploid cells.

## Results

### Induction of aneuploidy in early mouse embryos

**Figure 1:**
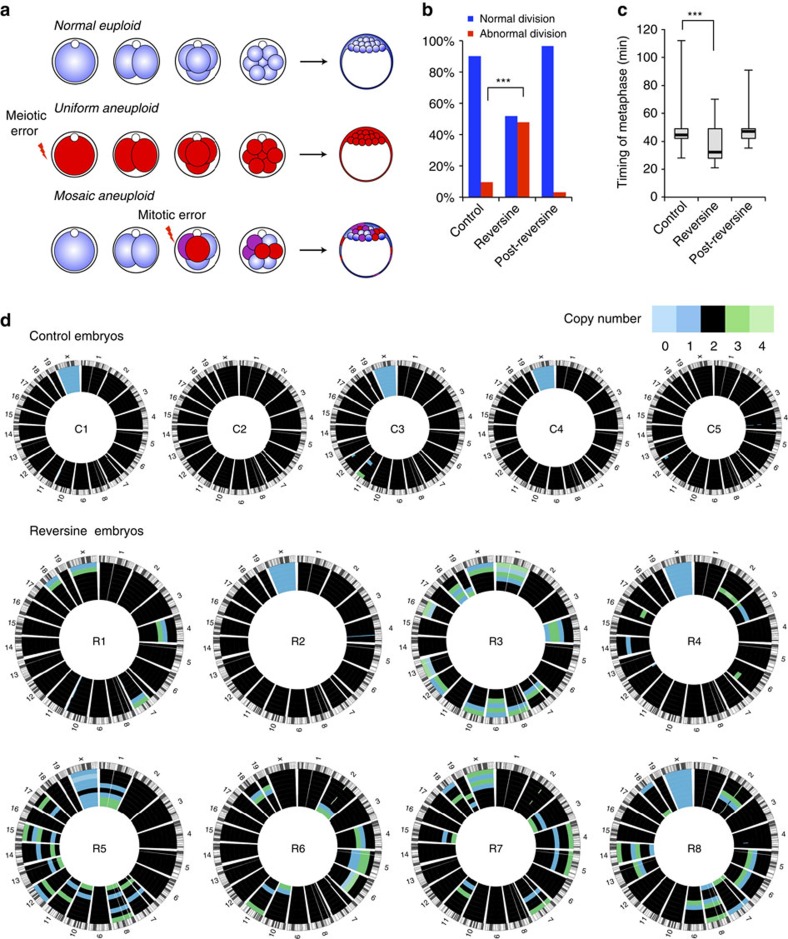
Reversine treatment induces aneuploidy in mouse embryos. (**a**) Uniform aneuploid embryos contain a single identical abnormality in their chromosome status and mosaic aneuploid embryos contain blastomeres with differing chromosome constitutions caused by mitotic error post fertilization. (**b**) Blastomeres treated with reversine during division (*n*=81) have significantly greater rates of chromosome missegregation during mitosis than controls (*n*=72; ***P<0.001, *χ*^2^-test). (**c**) Blastomeres treated with reversine have a shorter metaphase than controls (***P<0.001, Mann–Whitney *U*-test). These effects are reversible on washout of the drug (*n*=32). (**d**) Validation of aneuploidy in reversine-treated embryos, by single-cell genome sequencing. Each circos plot represents the genomic constitution of an embryo in all its cells, with blastomeres presented as rings and the chromosomes as segments. All results of the single-cell genome sequencing are provided in Supplementary Data 1.

To induce chromosome segregation errors in early pre-implantation mouse embryos (Fig. 1a) we treated embryos with reversine^14^, a small molecule inhibitor of Monopolar spindle 1-like 1 kinase, to inactivate the spindle assembly checkpoint (SAC). The effects of reversine are reversible following removal of the drug^14^; therefore, the embryos were treated with 0.5 μM reversine during the four- to eight-cell division, before being cultured in inhibitor-free medium until the mature blastocyst stage (E4.5). We found that this treatment had no effect on blastocyst formation, with a comparable percentage of reversine-treated embryos (93%, *n*=30) and controls (92%, *n*=36) developing into morphologically normal blastocysts. To confirm that the SAC was inactivated by reversine treatment, we co-incubated embryos with 0.33 μM nocadazole during the four- to eight-cell stage transition. All control embryos underwent prolonged arrest before their blastomeres eventually fragmented, with none dividing to the eight-cell stage (Supplementary Table 1, *n*=22). In contrast, 13 out of 26 embryos (50%) treated with 0.5 μM reversine were able to divide to the eight-cell stage and this increased to 17 out of 26 embryos (65%) with 1.0 μM reversine. To determine the effects of reversine treatment on chromosome segregation and the length of mitoses, embryos expressing Histone H2B-GFP^15^ were treated with 0.5 μM reversine and their development visualized by live imaging during the 8- to 16-cell division. Major chromosome segregation errors, such as lagging chromosomes and the formation of micronuclei, were found to occur during mitosis in 48% of reversine-treated blastomeres (*n*=81), compared with only 9.7% of control blastomeres (*n*=72, *P*<0.001, *χ*^2^-test; Fig. 1b). The length of metaphase was also significantly shorter in reversine-treated blastomeres (32.2 min) compared with controls (44.4 min, *P*<0.001, Mann–Whitney *U*-test; Fig. 1c). As expected, these effects were reversible. Following washout of reversine from the medium, the chromosome missegregation rate (3.1%) and length of metaphase (47.2 min) both returned to normal levels (*n*=32; Fig. 1b,c). These results demonstrate that reversine is an effective and reversible inhibitor of the SAC in pre-implantation embryos.

We used two independent methods to confirm that 0.5 μM reversine treatment during the four- to eight-cell stage transition generated aneuploid cells. First, we analysed control and reversine-treated embryos for their chromosome constitution at the early blastocyst stage (E3.5) using fluorescent *in situ* hybridization (FISH)^16^ for three randomly selected chromosomes: 2, 11 and 16. We found that reversine-treated blastomeres (*n*=1,076, 43 embryos) had significantly higher rates of aneuploidy than control blastomeres (*n*=1,376, 38 embryos), with abnormalities detected in 35% of reversine-treated blastomeres compared with 17% of controls (*P*<0.001, *χ*^2^-test; Supplementary Fig. 1). To obtain a more comprehensive, genome-wide view of the aneuploidy induced by reversine treatment, we applied low-pass single-cell genome sequencing to blastomeres isolated from eight-cell stage mouse embryos (Fig. 1d and Supplementary Data 1). Using genomic bins of 1 Mb for single-cell DNA copy number profiling by focal sequence-read depth analysis, individual cells from control (2 out of 37 cells) and reversine-treated (3 out of 59 cells) embryos had similarly low numbers of segmental rearrangements. However, whole chromosome missegregations were dramatically increased in reversine-treated cells (39 out of 59 cells, 66.1%), compared with controls (0 out of 37 cells, *P*<0.001, Fisher's test). Both approaches therefore demonstrate high levels of aneuploidy following reversine treatment, supporting the application of this technique for the generation of aneuploid blastomeres for use in a mouse model of chromosome mosaicism.

### Decreased cell number in aneuploid pre-implantation embryos

**Figure 2:**
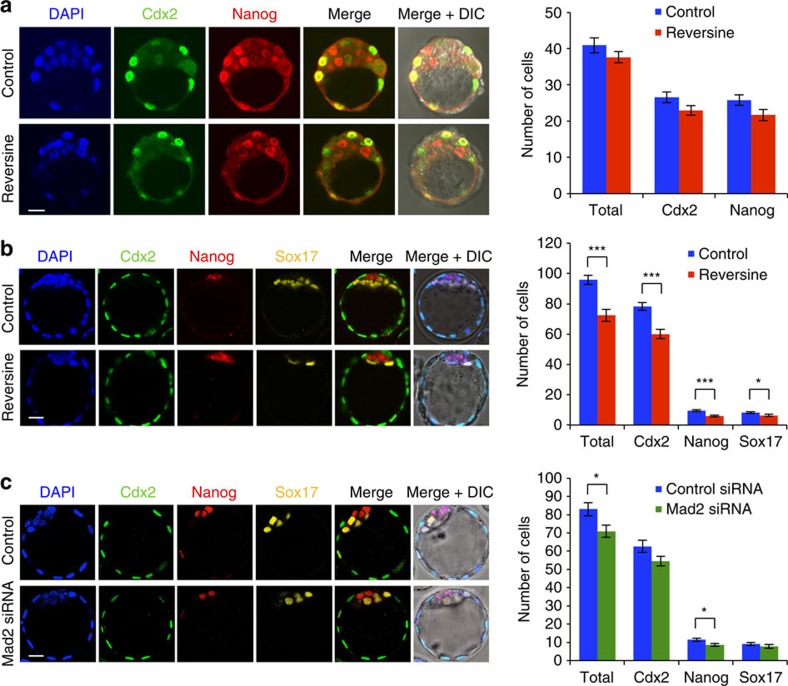
Preimplantation development of aneuploid embryos. (**a**) Embryos were treated with reversine (*n*=20) and analysed for expression of lineage-associated genes at the early blastocyst stage, compared with controls (*n*=24). No difference in the number of Cdx2-positive and Nanog-positive cells was observed. (**b**) Embryos were treated with reversine (*n*=26) and analysed for lineage specification at the expanded blastocyst stage, compared with controls (*n*=31). Reversine-treated embryos have a significantly reduced cell number in all lineages (*P<0.05 and ***P<0.001; Student's *t*-test), but the segregation of cell lineages was not affected. (**c**) Embryos were injected with Mad2 siRNA (*n*=22) or control siRNA (*n*=20) and analysed at the expanded blastocyst stage. Mad2 siRNA-injected embryos had significantly fewer cells than control siRNA-injected embryos (*P<0.05; Student's *t*-test), but this effect was not as clear as that seen with reversine treatment. Scale bars, 20 μm. Error bars represent s.e.m.

We next sought to determine the effects of reversine-induced aneuploidy on pre-implantation development. To this end, embryos were treated with 0.5 μM reversine during the four- to eight-cell stage transition and cultured to either the early (E3.5) or late (E4.5) blastocyst stage to examine the expression of markers of the TE (Cdx2), EPI (Nanog) and PE (Sox17; Fig. 2a,b). We found that at the early blastocyst stage there was no difference in total cell number between reversine-treated (37.6 cells, *n*=20) and control embryos (40.9 cells, *n*=24), and no difference in the number of Cdx2 and Nanog-positive cells (Fig. 2a). At the late blastocyst stage, however, the reversine-treated embryos had significantly fewer cells (72.3 cells, *n*=26) than controls (95.9 cells, *n*=31, *P*<0.001, Student's *t*-test; Fig. 2b). When all individual cell lineages were analysed, a significantly reduced average cell number was seen in the TE (60.0 compared with 78.2 cells, *P*<0.001, Student's *t*-test), EPI (5.9 cells compared with 9.4 cells, *P*<0.001, Student's *t*-test) and PE (6.4 cells compared with 8.3 cells, *P*<0.05, Student's *t*-test) in reversine-treated embryos. However, despite this reduction in cell number, all three lineages were segregated normally (Fig. 2b) and embryo morphology appeared unperturbed. To investigate whether the depleted cell number observed in the reversine-treated embryos could be caused by an off-target effect of reversine, we sought to inhibit the SAC by an alternative method. To this end, we injected zygotes with small interfering RNA (siRNA) targeting the SAC protein, Mad2 (refs 17, 18). We confirmed Mad2 downregulation and cultured embryos to the late blastocyst stage (Fig. 2c and Supplementary Fig. 2). We found that embryos injected with Mad2 siRNA (*n*=22) had a significantly depleted total cell number, compared with embryos injected with control siRNA (*n*=20, 70.9 cells compared with 83.0 cells, *P*<0.05, Student's *t*-test), with a significant decrease in the number of EPI cells (8.6 cells compared with 11.4 cells, *P*<0.05, Student's *t*-test). Therefore, Mad2 siRNA-injected embryos had a similar phenotype to that seen with reversine treatment; however, the effect was less strong, reflecting the inefficiency of RNA interference relative to drug treatment, leading us to use 0.5 μM reversine treatment as a means to induce aneuploidy in all subsequent experiments.

### Postimplantation lethality of aneuploid embryos

Our results demonstrated that reversine-treated embryos can establish the first three lineages, albeit with reduced cell numbers compared with controls; therefore, we next investigated whether these embryos could implant successfully and give rise to pups. To this end, we treated embryos with reversine, as before, and then transferred them at the blastocyst stage to recipient foster mothers, before recovering them at early postimplantation stages (E6.5–E7.5). We found that implantation rates were significantly higher in control embryos (71%, *n*=45) compared with those treated with reversine (50%, *n*=40, *P*<0.05, *χ*^2^-test; Table 1). Importantly, the reversine-treated embryos failed to produce any viable embryos and all implantation sites were resorbing. In contrast, every implantation site in the control embryos contained a viable embryo (*P*<0.001, Fisher's test; Table 1). These results indicate that despite morphologically normal pre-implantation development, reversine treatment ultimately results in early postimplantation lethality.

### Depletion of aneuploid cells before implantation

**Figure 3:**
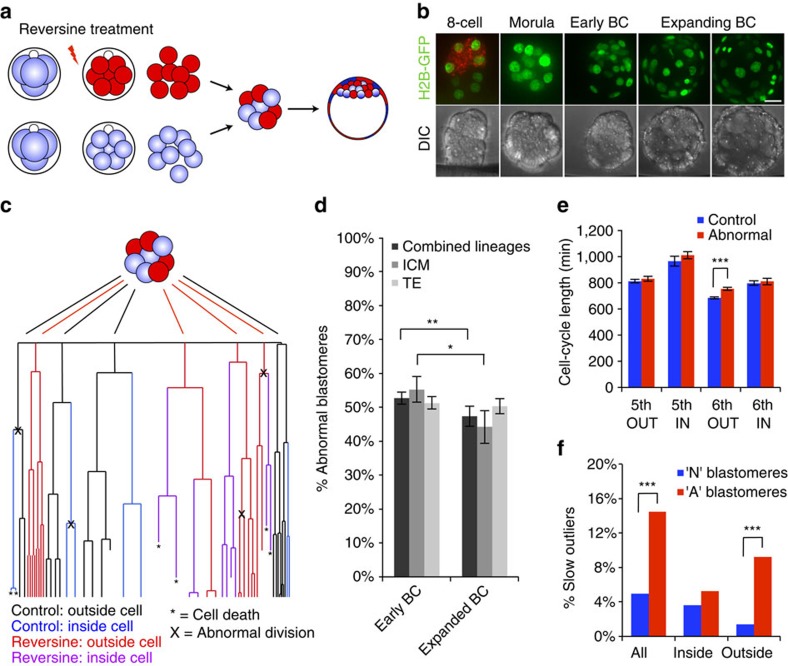
Pre-implantation development of chimeric mosaic embryos. (**a**) Chimeras containing a 1:1 ratio of control and reversine-treated blastomeres were generated at the eight-cell stage. (**b**) Chimeras expressing Histone H2B-GFP (*n*=20) were imaged for 2 days to the late blastocyst (BC) stage and the movements and divisions of all cells were tracked (*n*=1,079 cells). Scale bar, 20 μm. (**c**) Example of lineage tree showing the fate of control and reversine-treated blastomeres in a chimeric embryo. Cell tracking data are provided in Supplementary Data 2. (**d**) The number of abnormal cells decreases in the ICM during blastocyst expansion (*P<0.05 and **P<0.01; Student's *t*-test). (**e**) Average lengths of the fifth and sixth cell cycles for control and abnormal cells in inside and outside positions. Outside cells have a significantly longer sixth cell cycle than controls (***P<0.001; Student's *t*-test). (**f**) The proportion of slow outliers is greatly increased in the outside cells of the abnormal clone, compared with controls (***P<0.001, *χ*^2^-test). Error bars represent s.e.m.

To generate mosaic embryos that contain both aneuploid and euploid cells, we created aggregation chimeras at the eight-cell stage that contained a 1:1 ratio of reversine-treated and control blastomeres (Fig. 3a). Before chimera aggregation, one group of blastomeres was labelled with the red fluorescent dye FM4-64 so that each clone of cells could be identified at the start of imaging. The embryos were imaged for 48 h from the late eight-cell stage to the mature blastocyst stage and every cell in each embryo was tracked, using Histone-GFP fluorescent signal to visualize individual nuclei (*n*=20 embryos, 1,079 blastomeres; Fig. 3b). To ensure that any differences in blastomere behaviour could be attributed solely to their origin (control or reversine treated), an equal number of embryos in which either the control blastomeres or the reversine-treated blastomeres were labelled were tracked and used for analysis. To reveal the origin, history, cell behaviour and ultimate fate of each blastomere we generated cell lineage trees (Fig. 3c and Supplementary Data 2). Although some chromosome segregation errors are known to arise during normal development, these occurred at a very low and similar frequency in both reversine-treated (4.0%) and control blastomeres (5.0%), again demonstrating the reversibility of the effects of reversine treatment on SAC function. To take account of these errors, blastomeres demonstrating chromosome segregation errors in the control clone and their progeny were considered in the same group as the reversine-treated blastomeres (abnormal clone). We found that the average proportion of blastomeres originating from the abnormal clone in each embryo was 53% at the early blastocyst stage, but decreased to 47% by the mature blastocyst stage (*P*<0.01, Student's paired *t*-test; Fig. 3d). Detailed analyses revealed that this decrease was primarily due to a decrease in the proportion of abnormal cells in the ICM (from 55.8 to 44.2%, *P*<0.05, Student's paired *t*-test), whereas the decrease in the TE lineage was not significant (from 51.3 to 50.3%). Differences between the contribution of abnormal cells to the TE or ICM could not be accounted for by preferential allocation of these cells to the TE, as there was no difference in the pattern of cell division at the 8- to 16-cell and 16- to 32-cell transitions between abnormal (60% symmetric) and control (61% symmetric) blastomeres (*n*=361 divisions).

To determine whether differences in cell cycle lengths or cell cycle arrest could account for the depletion of blastomeres from the abnormal clone of the embryo, we measured the cell cycle lengths for all fifth and sixth cleavages at the 16- to 32-cell and 32- to 64-cell transitions (Fig. 3e). Mean cell cycle lengths were compared between control and abnormal clones, matched to cleavage cycle and according to position within the embryo: inside (ICM progenitors) or outside (TE progenitors). There were no significant differences between the control and abnormal clones, with the exception of outside cells undergoing the sixth cleavage. In this case, there was a small but significant increase in the mean cell cycle length in the abnormal clone of blastomeres (751 min, *n*=126 cells), compared with controls (681 min, *n*=142 cells, *P*<0.001, Student's *t*-test). This assessment of cell cycle lengths required the blastomeres to have completed a minimum of two divisions during imaging, meaning that extremely slow or senescent cells were excluded. To include these cells in the analysis, blastomeres with unmeasurable cell cycle lengths were assessed up until the point at which the movie ended. All blastomeres were then categorized as either ‘standard' or ‘slow outliers' depending on whether their cell cycle length was less or more than the mean cell cycle length plus two s.d., respectively. Blastomeres that had unmeasurable cell cycle length but did not exceed the cutoff limit for categorization as slow outlier were excluded, as it was not possible to determine which category they belonged to. Using this classification system we found that a significantly higher proportion of blastomeres in the abnormal clone were slow outliers (13.5%) than in the control clone (5.0%, *P*<0.001, *χ*^2^-test; Fig. 3f). When TE and ICM cells were analysed individually, this effect was specific to the TE, with 9.2% of TE cells from the abnormal clone being slow outliers, compared with only 1.4% of TE cells from the control clone (*P*<0.001, *χ*^2^-test). In the ICM, the difference in proportion of slow outliers was small and not statistically significant (5.2% and 3.6% in abnormal and control clones, respectively). When considered together, the trend towards slower cell cycle lengths, combined with the higher proportions of slow outlier cells in the abnormal TE cells, could account for a proportion of the reduction in abnormal cell number observed in the chimeric embryos.

### Apoptosis of aneuploid cells during blastocyst maturation

**Figure 4:**
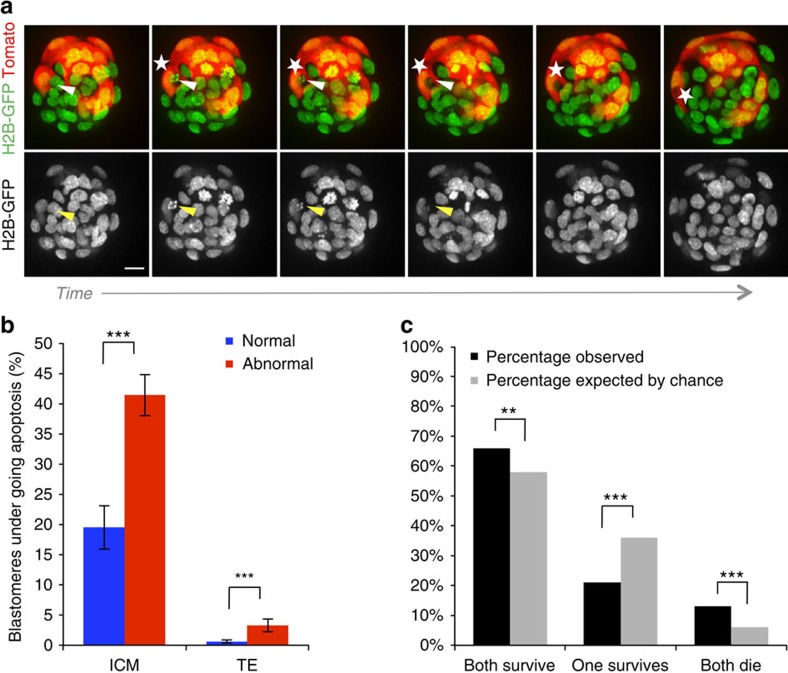
Elimination of abnormal cells by apoptosis during blastocyst development. (**a**) Sequential images from a time-lapse series of a 1:1 reversine chimera (control cells in this embryo are labelled with Tomato fluorescent protein), showing apoptosis of a reversine-treated cell (arrow), followed by engulfment of the apoptotic debris into an efferosome by a neighbouring control cell (star). Scale bar, 20 μm. (**b**) The percentage of blastomeres undergoing apoptosis is significantly increased in the abnormal clone (*n*=36 embryos, 634 ICM cells, 1,346 TE cells; ***P<0.001; Fisher's test) Error bars represent s.e.m. (**c**) Sister blastomeres (*n*=321 pairs) are more likely to have similar fates than would be expected by chance (**P<0.01 and ***P<0.001; binomial test).

In addition to differences in cell cycle lengths, a further mechanism that may be responsible for the gradual depletion of abnormal cells is an increased frequency of apoptosis. Apoptosis is frequently observed within the ICM lineage during blastocyst maturation; therefore, in addition to the 20 chimeras that were analysed from the eight-cell stage onwards, a further 14 embryos were included for the analysis of apoptosis, to increase the number of ICM cells that could be assessed. These chimeras were generated using embryos that had been injected with *Tomato-RFP* messenger RNA into both blastomeres at the two-cell stage, removing the requirement for FM4-64 labelling, and were imaged for the 24-h period encompassing blastocyst maturation (Supplementary Data 2). We detected characteristic apoptotic morphological features^19^ in 30.9% of the ICM cells of chimeric embryos (*n*=36 embryos, 634 blastomeres). This included the disintegration of apoptotic cells and the subsequent engulfment of the cellular debris by neighbouring cells (Fig. 4a). We found that frequency of apoptosis in the ICM was significantly higher in the abnormal clone of cells (41.4%) than the control clone (19.5%, *P*<0.001, Fisher's test; Fig. 4b), indicating that aneuploid cells are preferentially eliminated from the embryo. In the TE, apoptosis was much less frequent (2.0% of cells, *n*=1346 blastomeres), but again the abnormal clone showed a significantly higher frequency of apoptosis (3.3%) than the control clone (0.6%, *P*<0.001, Fisher's test; Fig. 4b).

The higher rate of apoptosis observed within the abnormal clones suggested that genetically abnormal cells might be eliminated from the future fetal lineage by apoptosis. If this is the case, it would follow that cells with the closest common ancestry would be likely to share a common fate, either to all die or all survive. To test this hypothesis, we next evaluated the patterns of cell death or survival in ICM sister blastomere pairs, which are the direct progeny of one parent blastomere and thus more likely to share identical genetic constitutions (*n*=321 pairs; Fig. 4c). We found that significantly more sister blastomere pairs both underwent apoptosis than would be expected by chance (13.3 versus 5.6%, *P*<0.001, binomial test). Furthermore, more pairs of blastomeres both survived than would be expected if apoptosis occurred randomly (66.0% versus 58.2%, *P*<0.01, binomial test). These results provide evidence that abnormal cells are actively eliminated from the ICM of chimeric mosaic embryos by apoptosis during blastocyst development.

### Presence of sufficient euploid cells rescues development

To address whether the presence of normal cells within the embryo could rescue the lethal peri-implantation phenotype of reversine-induced aneuploidy, we next investigated the postimplantation development of mosaic embryos. Chimeras containing a 1:1 ratio of control and reversine-treated blastomeres were constructed at the eight-cell stage, as before (*n*=115). In addition, chimeras were also made with a 1:3 ratio of control to reversine-treated blastomeres (*n*=53). The embryos were transferred to foster mothers and recovered at early postimplantation stages (E6.5–E7.5; Table 1). We found that both the 1:1 chimeras (64.3%, *P*=0.09, *χ*^2^-test) and the 1:3 chimeras (47.2%, *P*=0.47, *χ*^2^-test) had equivalent implantation rates to control chimeras (53.3%, *n*=107). Strikingly, all 1:1 chimera implantation sites contained a viable, morphologically normal embryo, as did controls (Table 1), indicating that the addition of control blastomeres in the 1:1 chimeras was able to rescue the reversine phenotype. In contrast, significantly fewer of 1:3 chimera implantation sites contained a viable embryo (44%, *P*<0.001, Fisher's test; Table 1), although there was still a partial rescue of postimplantation lethality. No evidence of increased apoptosis was found in either control or 1:1 reversine chimeras by TdT-mediated dUTP nick end labelling (TUNEL) staining (not shown), indicating that apoptosis is unlikely to play a major role in eliminating abnormal cells during early postimplantation development.

**Figure 5:**
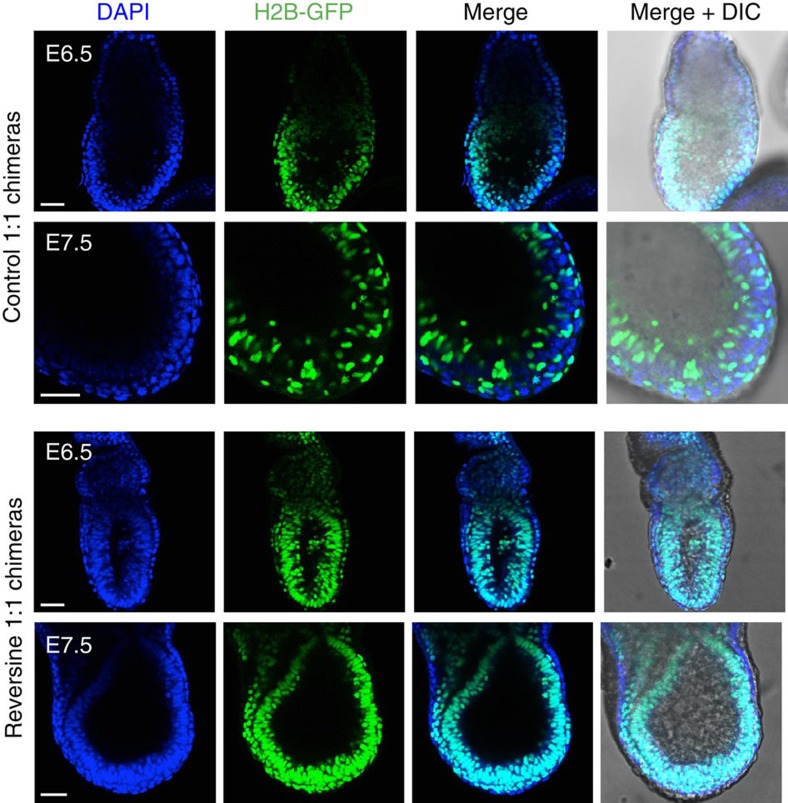
Loss of reversine-treated cells in postimplantation chimeric mosaic embryos. Chimeras that had been transferred to recipient females were recovered at early postimplantation stages (E6.5–E7.5) and the contribution of reversine-treated cells to the egg cylinder assessed. In these examples, the control clone is marked by the expression of Histone H2B-GFP. In controls, both clones contribute to the embryo. In the reversine chimeras, the majority of each embryo originates from the control clone (demonstrated by the lack of DAPI-positive/GFP-negative cells). Scale bars, 50 μm.

In a subset of the experiments, 1:1 reversine and control chimeras were constructed using embryos from two different genetic backgrounds, wild-type F1 and Histone H2B-GFP, so that the control and reversine-treated clones could be identified in the postimplantation embryos. The composition of these chimeras was analysed at E6.5 and E7.5 (Fig. 5). We found that in the majority of embryos recovered, there were dramatically fewer reversine-treated cells than control cells; however, some reversine-treated clones were present throughout the entire conceptus, with no evidence of preferential depletion in the embryonic or extra-embryonic lineage. It is possible that these clones represented reversine-treated blastomeres that did not carry chromosomal abnormalities. There was also no evidence of increased apoptosis or necrosis occurring in the treated clones, as nuclear morphology appeared equivalent between the reversine-treated and control clones, with no evidence of nuclear debris or cellular remnants. These findings suggest that although abnormal cells are depleted from the embryo before E6.5, the reversine-treated clones that remain contribute to both embryonic and extra-embryonic tissues.

### Development of mosaic embryos to term

**Figure 6:**
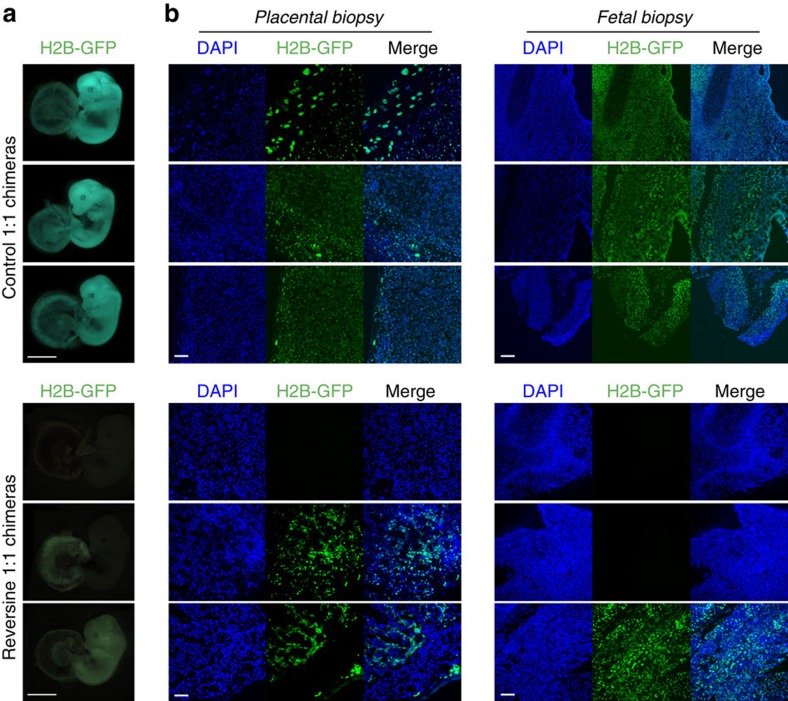
Successful fetal development of chimeric mosaic embryos. Chimeras that had been transferred to recipient females were recovered at E13.5. (**a**) Examples of whole-mount images (Histone H2B-GFP expression marks the reversine-treated clone). Scale bars, 2 mm. Note: the fetuses were imaged using different microscopes, the difference in colour is reflective of the microscope used, not differences between the two groups. (**b**) Fetal and placental biopsies from the E13.5 conceptuses shown in A. Scale bars, 100 μm. In all cases, where a Histone H2B-GFP clone was present, the cells were evenly distributed throughout the tissues. Both placental and fetal biopsy findings were consistent with the whole-mount GFP images.

The above experiments indicated that the early postimplantation lethality associated with aneuploidy could be rescued, provided there were a sufficient number of normal cells present within the embryo. Therefore, we next sought to determine whether this early postimplantation rescue was associated with successful further development. To investigate this, 1:1 reversine chimeras and equivalent control embryos were transferred into foster mothers and recovered at E13.5 or allowed to develop to term. We found that chimeras comprising half reversine-treated cells developed to E13.5 with an equivalent rate (52.3%, *n*=58) to controls (58.8%, *n*=34, *P*=0.53, *χ*^2^-test; Table 1), with the appropriate size and morphology expected for their gestational stage. To evaluate whether there was any significant depletion of the reversine-treated clone within the fetus or placenta, we also generated chimeras from a mix of wild-type F1 and Histone H2B-GFP embryos. On recovery of the conceptuses at E13.5, whole-mount green fluorescent protein (GFP) images were taken (Fig. 6a), along with fetal and placental biopsies (Fig. 6b). In the majority of control chimeras, both clones were present in both the fetus and placenta (88%, *n*=17 embryos). In the chimeras containing reversine-treated cells, these cells were depleted or absent from the fetus in 66% of embryos (*n*=9, *P*<0.01 compared with controls, Fisher's test) and from the placenta in 55% of cases (*n*=9, *P*<0.01 compared with controls, Fisher's test). In those cases where both clones were present within the conceptus, evaluation of the biopsies revealed that both clones were distributed evenly and mixed, with no evidence of clonal pooling or restrictions. There was also no evidence of enrichment of the abnormal clone within the placental tissues.

Finally, to determine whether viability rates at E13.5 would be consistent with live birth rates, we transferred chimeras containing 50% reversine-treated cells to recipient mothers and allowed their development to term (*n*=26). A total of 13 live pups were born, confirming that viability at E13.5 was indeed equivalent to viability at term (*P*=0.77, *χ*^2^-test; Table 1 and Supplementary Fig. 3). All pups survived with no signs of ill health beyond 4 months of age (day 3 mean weight=3.31 g, s.d.=0.26 g; Supplementary Table 2). Analyses of coat fur revealed no evidence of the reversine-treated clone in 7 out of 13 mice, with only 3 mice having a major contribution from both clones. Thus, in the minority of embryos that contained progeny from the reversine-treated clone, there was no evidence of adverse outcome. Together, these results show that the embryonic lethality of reversine-treated embryos can be completely rescued, provided there are sufficient normal cells present in the embryo. The combined E13.5 recovery and live birth rates were equivalent to early postimplantation viability rates (*P*=0.09, *χ*^2^-test; Table 1), suggesting that successful implantation equated to the complete restoration of developmental potential.

## Discussion

Here we generated a mouse model for euploid–aneuploid chromosome mosaicism, to investigate both the fate of aneuploid cells and the developmental potential of mosaic embryos. To induce aneuploidy, we used the drug reversine to inhibit the key SAC protein Monopolar spindle 1-like 1 kinase during the four- to eight-cell stage transition^14^. Reversine treatment successfully achieved bypass of the SAC and induced high rates of chromosome segregation errors in treated blastomeres. In addition, the effect of the treatment was transient and SAC function was restored on washout of the drug, enabling the induction of acute chromosome missegregations to be tightly restricted and thus limited to a defined developmental window, similar to that believed to occur during human pre-implantation development^1^. Reversine-treated embryos formed blastocysts by E4.5, but had significantly fewer cells in all lineages, compared with controls. We also attempted to inhibit SAC function in the mouse embryo using siRNA targeting another SAC component, Mad2 (ref. 17). Similar to reversine-treated embryos, Mad2 siRNA-injected embryos had a reduction in cell number at E4.5, although this effect was smaller, most probably reflecting the inefficiency of RNA interference relative to drug treatment. The similarity of phenotypes suggests that the reduced cell number is not an off-target effect of reversine, but rather represents a true phenotype downstream of SAC inhibition.

Despite morphologically normal development to the blastocyst stage, all reversine-treated embryos died by early postimplantation stages, reminiscent of the early postimplantation lethal phenotype of embryos lacking essential SAC genes or kinetochore proteins^17^,^20^,^21^,^22^,^23^,^24^,^25^,^26^. Embryos that lack expression of BubR1, for example, display mosaic aneuploidy, are lethal at early postimplantation stages and exhibit high levels of apoptosis^26^. These SAC knockout embryos, which are in a state of permanent chromosome instability, have shown that SAC function is essential for embryo development. Here, by using a reversible inhibitor during only one early division, we have generated aneuploid embryos that have normal SAC function following removal of reversine from culture, and therefore chromosome content should be stable, with the aneuploidies generated by the reversine treatment maintained through subsequent division cycles. This increases the likelihood that the peri-implantation lethality of reversine-treated embryos is due to reversine-induced aneuploidy, rather than other effects of long-term SAC inhibition and chromosome instability.

To generate mosaic embryos in which normal and abnormal cells could be identified, we created chimeric mouse embryos containing a 1:1 ratio of control and reversine-treated cells. The use of wild-type cells for the ‘normal' part of the chimera was considered appropriate as, unlike in human embryos, the prevalence of aneuploidy in mouse embryos is relatively low^27^,^28^,^29^, which we confirmed by our single-cell genome sequencing of control eight-cell stage embryos. To determine the fate of abnormal cells within developing mosaic chimeras, we used high-resolution time-lapse imaging to follow every single cell within each embryo. Although current technology did not enable the direct evaluation of the chromosome status in each blastomere, a comparative approach to analysis was adopted, on the basis that reversine-treated blastomeres with high rates of chromosome segregation errors would be expected to exhibit greater rates of chromosome abnormalities than their control counterparts. Importantly, we found that between E3.5 and E4.5 the abnormal clone of cells was significantly depleted from the embryo, in agreement with observations in human embryos that identified the proportion of aneuploid cells to be lower in blastocysts than at earlier developmental stages^30^,^31^,^32^,^33^,^34^,^35^. These data are consistent with a ‘clonal depletion' hypothesis^35^,^36^ for the survival of mosaic embryos, rather than the ‘self-correction' of chromosomal abnormalities^37^,^38^.

The application of time-lapse imaging enabled cell fate to be evaluated directly in mosaic embryos for the first time and revealed no evidence of preferential allocation of abnormal cells to embryonic or extra-embryonic lineages, in agreement with studies on human embryos^3^,^33^,^34^,^39^,^40^. Instead, we found that in different lineages different mechanisms are primarily responsible for the depletion of reversine-treated cells, with TE cells exhibiting increased cell cycle length and senescence, and ICM cells having an increased frequency of apoptosis. Whether this apoptotic response in the ICM is downstream of aneuploidy-induced DNA damage or another pathway is not yet clear; however, it is in agreement with previous models of chromosome instability that reported cell death within the ICM lineage, in contrast to reduced but ongoing viability of TE cells^17^,^20^,^23^. The evidence we provide here against the hypothesis that aneuploid cells are preferentially allocated to the TE does not exclude the possibility that confined placental mosaicism^41^ may arise as a consequence of pre-implantation defects. We found that abnormal cells within the ICM were significantly more likely to undergo apoptosis than those within the TE. Hence, abnormal TE cells often remained viable, albeit depleted relative to their normal counterparts. If some of these cells continue to proliferate, contributing progeny to the future placenta, this could result in confined placental mosaicism.

To our knowledge, this is the first study to directly demonstrate the progressive depletion of aneuploid cells from the pre-implantation embryo, and to establish that this occurs through differential mechanisms according to cell lineage. This is also the first study to provide direct evidence that apoptosis within the ICM can occur as a mechanism to eliminate cells with chromosomal abnormalities, a hypothesis frequently proposed^42^,^43^ but until now never directly tested. To take this further and determine the postimplantation fate of euploid–aneuploid mosaic embryos, we transferred the experimental chimeras into foster mothers. This revealed that when embryos contained 50% non-treated control cells, the early postimplantation lethality that occurred in whole reversine-treated embryos was completely rescued and implantation rates and viability were comparable to those in equivalent controls. When the proportion of control blastomeres was decreased to one third, there was a partial rescue of embryonic lethality. Unlike in postimplantation embryos lacking key components of the SAC, such as Mad2 (ref. 17) or BubR1 (ref. 26), we did not observe widespread apoptosis in the 1:1 reversine chimeras at E6.5–E7.5, again most probably because although the chimeras contain a high proportion of abnormal cells, the SAC is functional in these embryos.

**Figure 7:**
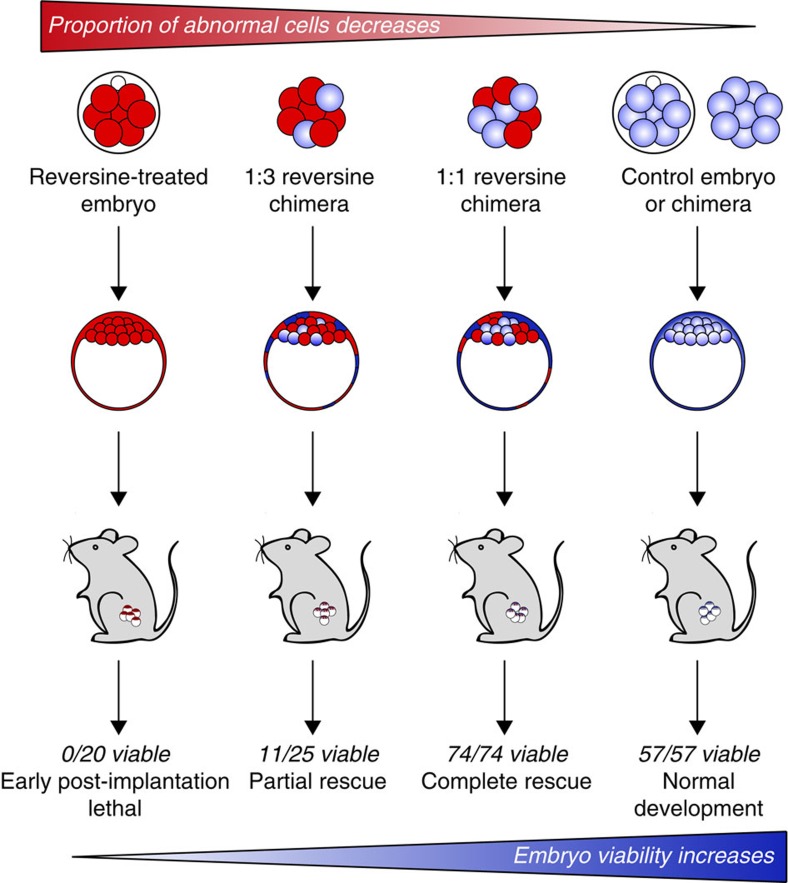
Effects of pre-implantation chromosome mosaicism on embryo development and survival. Reversine-treated embryos formed blastocysts but failed to develop past implantation. Increasing the proportion of control blastomeres in the embryo rescued the lethal phenotype. Numbers represent the viability of early postimplantation embryos that had successfully implanted. Early postimplantation rescue resulted in complete rescue of developmental potential, with E13.5 and live birth rates equivalent to viability at E6.5–7.5.

At the early postimplantation stages of development, many 1:1 chimeric embryos still contained a small proportion of reversine-treated progeny, but by E13.5 this was not the case in the majority of fetuses and their placenta. It is likely to be that an ongoing reduction in the abnormal clone relative to the control clone occurs due to poorer proliferative capabilities of the abnormal cells. When these 1:1 chimeras were allowed to develop to term, live pups were born with comparable success to early postimplantation and fetal viability rates. Together, these results lead us to conclude that euploid–aneuploid mosaic embryos are likely to be viable, provided there is a sufficient proportion of normal cells within the embryo, and that viability at the early postimplantation stage of development is predictive of survival to fetal stages and live birth (Fig. 7). This finding may be of clinical significance when viewed in the context of embryo biopsy and pre-implantation genetic screening, and potentially an explanation for why cleavage stage embryo biopsy was found to be detrimental to IVF success rates in couples with a poor prognosis for IVF^44^. It also supports the current move from biopsy at the cleavage stage towards blastocyst biopsy, when removal of cells has less impact on the overall constitution of the embryo.

In conclusion, the results presented here show for the first time that blastomeres with a history of chromosome abnormalities become progressively depleted from the embryo as it develops. This depletion becomes first apparent during blastocyst maturation, when abnormal ICM cells have increased apoptosis and abnormal TE cells exhibit limited proliferation. Abnormal embryos are able to implant and elicit a maternal decidual reaction, but undergo early postimplantation resorption. In contrast, mosaic embryos that contain sufficient normal euploid cells have full developmental potential.

## Methods

### Pre-implantation embryo culture and time-lapse imaging

Animals used for the study were maintained at the Gurdon Institute Animal Facility, University of Cambridge, with a 12:12 light cycle and provided with food and water *ad libitum*. All experiments were performed in compliance with Home Office regulations. Four- to six-week-old F1 (C57B16xCBA) female mice were injected with 10 IU PMSG (Intervet) followed by 10 IU hCG (Intervet) 48 h later, to induce superovulation. The females were then mated with F1 males or, where indicated, with Histone H2B-GFP males^15^. Embryos were recovered into M2 medium supplemented with 4 mg ml^−1^ BSA and cultured in KSOM medium with amino acids under mineral oil at 37 °C and 5% CO_2_. Reversine (Cayman Chemicals) was dissolved in dimethylsulfoxide and control embryos were incubated in the equivalent dimethylsulfoxide concentration. Nocodazole (Sigma) was diluted in culture medium. Embryos for live imaging were transferred to glass-bottom dishes (MatTek) and cultured within the individual interstices of a finely weaved nylon mesh. Time-lapse movies were generated using a spinning disk confocal microscopy system (3i Intelligent Imaging Innovations) and SlideBook software. Brightfield and fluorescent images were captured in 15 Z-planes at 3.5 μm intervals every 7–10 min. Individual cells were tracked using SimiBiocell software with the position of each nuclei marked in every frame. Cell tracking experiments were carried out blind to the origin of the blastomere, with identification of cell origin revealed only on completion of tracking of the entire embryo.

### Fluorescent *in situ* hybridization

FISH was carried out using probes for chromosomes 2, 11 and 16. Whole embryos were spread on poly-L-lysine slides and incubated for 20 min at 37 °C in 0.1 N HCl with 10 mg ml^−1^ pepsin (Sigma). After washes in H_2_O and PBS, the slides were fixed in 1% paraformaldehyde (PFA), washed with PBS and dehydrated through 70%, 90% and 100% ethanol for 3 min each. The slides and dual-colour LSI FISH probes (Kreatech, The Netherlands) were separately denatured at 75 °C for 5 min in 70 μl de-ionized formamide (Sigma), 20 μl H_2_O and 10 μl 20 × SSC, before being washed in cold 70% ethanol for 5 min and dehydrated through 90 and 100% ethanol for 3 min each. The probes were applied to the air-dried slides, incubated overnight at 37 °C and washed in 60% formamide/2 × SCC and 2 × SCC for 5 min each at 42 °C. The slides were then washed twice in 4 × SSX/0.05% Tween 20 for 5 min at room temperature, dehydrated and mounted using Vectashield (Vector Labs) containing 4,6 diamidino-2-phenylinodole (DAPI; Sigma). The slides were visualized using an Olympus BX40 microscope and Smartcapture II software (Digital Scientific).

### Single-cell genome sequencing

To collect individual blastomeres for single-cell genome sequencing, the zona pellucida of eight-cell stage embryos was removed by treatment with acidic Tyrode's (AT) solution (Sigma) and the embryos incubated in Ca^2+^ and Mg^2+^-free M2 media for 5 min. The embryos were then disaggregated into individual cells by gentle pipetting with a flame-polished glass pipette and each cell was snap frozen in PBS. The DNA of the single blastomeres was whole-genome amplified using PicoPlex (Rubicon Genomics). Sequencing libraries of whole-genome amplified products were prepared following the TruSeq or Nextera XT protocol (Illumina), which were performed according to the manufacturer's instructions with one exception: the Nextera XT protocol was performed using half volumes. The single-cell libraries were sequenced on the HiSeq2000 or HiSeq2500 platform and the resulting sequence reads were trimmed for potential adaptor contamination to specific read lengths, as specified in Supplementary Data 1, and then mapped to the mouse reference genome (mm9) using Burrows–Wheeler Alignment. Genome-wide DNA copy number profiles were generated using focal read-depth analysis. To this end, genomic bins of variable size, containing 1 million uniquely mappable positions (similar to^45^ and^46^) were defined. Specifically, we produced artificial reads of a length equal to the single-cell reads from every base in the mm9 reference genome and mapped them back to mm9 using Burrows–Wheeler Alignment, allowing the identification of all uniquely mappable positions in the mm9 reference genome using reads of a specific length. The reference genome was then divided into non-overlapping bins comprising the fixed number of uniquely mappable positions. Per bin, a single-cell logR value was then computed by determining the log2-base of the ratio of the cell's sequence read count within a given bin and the cell's median sequence read count of the bins genome wide. Bins with a %GC content of <28% were discarded. LogR values were corrected for %GC bias using a Loess fit in R and further normalized to the median logR value genome wide. Piecewise constant fitting was used to segment the logR values (the penalty *γ*-value was set to 15 and the kmin value to 5) and subsequently DNA copy number was estimated as 2^LogR^.Ψ with the average ploidy (Ψ) set to 2. Copy number analysis and statistical tests were performed using R (version 3.0.2; http://cran.r-project.org). The single-cell sequences had to fulfill quality criteria: a median absolute pairwise difference of the genome-wide logR of <0.4 or a minimum amount of sequencing reads obtained. See Supplementary Data 1.

### Immunofluorescence and TUNEL staining

Embryos were treated with AT solution to remove the zona pellucida and fixed in 4% PFA for 20 min at room temperature. The embryos were then permeabilized for 20 min in 0.6% Triton X-100 (Sigma), washed in PBS and incubated in blocking solution (3% BSA (Sigma) in PBS) at 4 °C for 4 h and then with primary antibodies in blocking solution for a minimum of 6 h at 4 °C. They were then washed and incubated with secondary antibodies for 1 h, washed again and incubated with DAPI for 5 min. Primary antibodies used: mouse anti-Cdx2 (1:200; BioGenex), rabbit anti-Nanog (1:200; Abcam) and goat anti-Sox17 (1:200; R&D Systems). For TUNEL staining, the embryos were fixed, washed and permeabilized for 30 min in 0.5% Triton-X100 on ice. The embryos were then washed in PBS/PVP (PBS containing 1 mg ml^−1^ polyvinylpyrrolidone (Sigma) and incubated in 50 μl of TUNEL reaction mixture (Sigma) in the dark for 1 h at 37 °C. Embryos were then washed through PBS/PVP and incubated in DAPI for 5 min at room temperature before imaging. Confocal imaging was carried out using either an Olympus FV1000 (Fluoview FV10-AW software) or Leica SP5 (LAS AF software) inverted confocal microscope. Image files were viewed and analysed using ImageJ (http://imagej.nih.gov/ij/) or Fiji (www.fiji.sc/wiki/index.php/Fiji) software.

### Injection of siRNA and confirmation of knockdown by qRT–PCR

Zygotes were injected with 12 μM *Mad2* (5′-GAGGACAGCTTTACTATTCAA-3′) or AllStars Negative Control (Qiagen) siRNA. Embryos were collected for quantitative reverse transcriptase–PCR (qRT–PCR) 48 h later at the eight-cell stage. Total RNA was extracted using the Arcturus PicoPure RNA Isolation Kit and qRT–PCR was performed using the Power SYBR Green RNA-to-CT 1-Step Kit (Life Technologies) and a StepOne Plus Real-time PCR machine (Applied Biosystems). The ddCT method was used to determine relative levels of mRNA expression, with *Gapdh* as an endogenous control. *Mad2* primers: 5′-CTGACCCCGAGCTCATAAAGT-3′, 5′-ACTGAGCACTTGTACAGCCA-3′ and *Gapdh* primers: 5′-AGAGACGGCCGCATCTTC-3′, 5′-CCCAATACGGC CAAATCCGT-3′.

### Generation of chimeric embryos and blastomere labelling

Aggregation chimeric mosaics containing reversine-treated and control blastomeres were created at the eight-cell stage. Individual blastomeres were obtained as described above and carefully aggregated together in M2 containing 0.1 mg ml^−1^ phytohaemagglutinin (Sigma). For live imaging from the eight-cell stage, the blastomeres were labelled just before chimera aggregation by incubation for 10 min in M2 containing a 1:100 dilution of FM4-64 (Invitrogen). For chimeras imaged from the mid-blastocyst stage, half of the embryos were labelled at the two-cell stage by microinjection of Tomato fluorescent protein mRNA into both blastomeres.

### Embryo transfers and postimplantation recovery and biopsy

Uterine transfers to prepared females were performed at the Transgenic Facility, Stem Cell Institute, University of Cambridge. E3.5 blastocysts were transferred into the uterine horn of 2.5 days postcoitum pseudo-pregnant F1 females that had been mated with vasectomized males. Early postimplantation embryos (E6.5–E7.5) were dissected from the maternal decidua and recovered into PBS on ice. Viable embryos were fixed in 4% PFA at 4 °C for 1 h, followed by washes through PBS and incubation with DAPI. E13.5 embryos were recovered individually into PBS on ice, keeping each fetus and its corresponding placenta together for whole-mount images. Placental and fetal biopsies were taken from several sites and fixed in 4% PFA for 1 h at 4 °C. The specimens were then rinsed in PBS and incubated sequentially in 15, 20 and 30% sucrose (Sigma) for 1 h each, followed by overnight incubation in a 1:1 30% sucrose:OCT embedding solution (Tissue-Tek) mix at 4 °C. The samples were then embedded in OCT media, frozen on dry ice and used to produce 12 μm cryosections. The slides were stained with NucView DAPI (Invitrogen) and mounted with Vectashield mounting medium (Vector Laboratories). Images were taken using a Deltavision widefield microscope with a × 20 objective.

### Statistical analysis

To compare categorical data the *χ*^2^-test was used, unless the numbers were small in which case Fisher's exact test was used. The Mann–Whitney *U*-test or Kruskall–Wallis test was used to compare median timings of mitosis or time to mitotic slippages. In all cases, the two-tailed version of the test was used to evaluate statistical significance. Calculations were carried out in Microsoft Excel or with GraphPad software. All graphs show mean values, error bars=s.e.

## Additional information

**Accession codes:** Single-cell genome sequences have been deposited in the European Nucleotide Archive (ENA) database under accession code PRJEB12768 (ERP014275).

**How to cite this article:** Bolton, H. *et al*. Mouse model of chromosome mosaicism reveals lineage-specific depletion of aneuploid cells and normal developmental potential. *Nat. Commun.* 7:11165 doi: 10.1038/ncomms11165 (2016).

## Supplementary Material

Supplementary Information Supplementary Figures 1-3 and Supplementary Tables 1-2

Supplementary Data 1 Single-cell genome sequencing

Supplementary Data 2 Chimera movie data

## Figures and Tables

**Table 1:** Summary of embryo transfer experiments.

	Early postimplantation (E6.5–E7.5)			E13.5		Live birth	
	Transferred	Viable	Non-viable	Transferred	Recovered	Transferred	Recovered
*Whole embryos*							
Control	45	32	0				
Reversine	40	0	20				
							
*Chimeras*							
Control	107	57	0	34	20		
1:1 Reversine	115	74	0	58	31	26	13
1:3 Reversine	53	11	14				
