# Supplementary material for: Mouse model of chromosome mosaicism reveals lineage-specific depletion of aneuploid cells and normal developmental potential
Source: Nat Commun. 2016 Mar 29;7:11165. doi: 10.1038/ncomms11165 (PMC4820631; doi:10.1038/ncomms11165)
Supplement: Supplementary Information — Supplementary Figures 1-3 and Supplementary Tables 1-2 [file ncomms11165-s1.pdf]

Supplementary Figures:

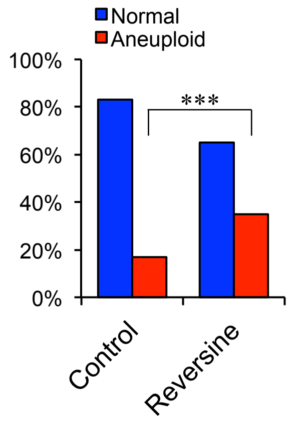

Supplementary Figure 1. Increase in aneuploidy following reversine treatment detected by FISH. Blastomeres were classified as normal or aneuploid based on FISH analysis for chromosomes 2, 11 and 16. Reversine-treated cells (n = 1076) had significantly higher rates of aneuploidy than controls (n = 1376; *** $p < 0.001$, χ^2)

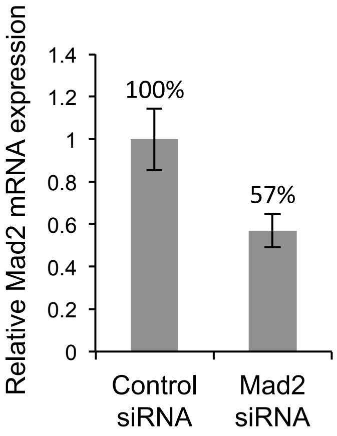

Supplementary Figure 2. Decreased Mad2 mRNA expression following zygote injection of Mad2 siRNA. Zygotes were injected with siRNA and mRNA expression assessed by RT-qPCR 48 hours later at the eight-cell stage (Mad2 siRNA n = 51 embryos, control siRNA n = 54 embryos, 4 independent experiments). Error bars = s.e.m.

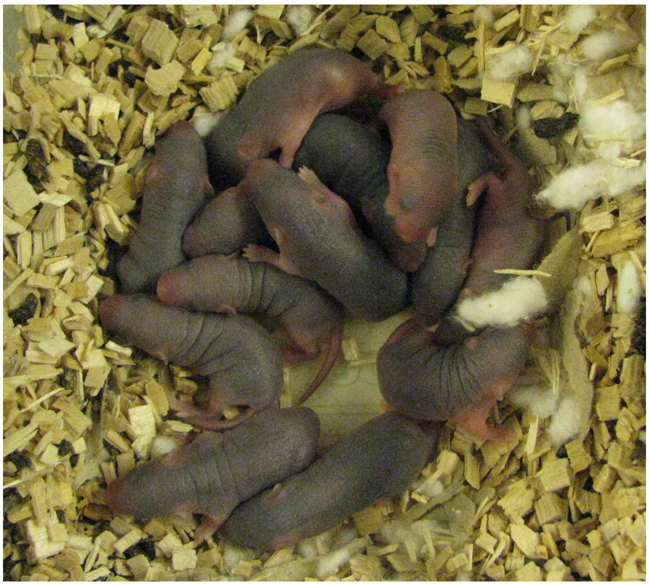

Supplementary Figure 3. Development of chimeric mosaic embryos to birth. 1:1 Reversine chimeras (n = 26) were transferred to foster mothers, resulting in the birth of 13 live pups.

Supplementary Tables:

Supplementary Table 1. Reversine-treated embryos bypass Nocodazole-induced metaphase arrest.

	**Arrest and fragment**	**Divide normally**	**Divided (total)**
**Control**	22	0	0% (22)
**Reversine 0.5µM**	13	13	50% (26)
**Reversine 1.0µM**	9	17	65% (26)

Supplementary Table 2. Newborn weights of chimeric mosaic pups.

**Pup**	**Weight (g)**
1	3.4
2	3.4
3	3.2
4	3.5
5	3.4
6	3.3
7	3.5
8	3.2
9	3.0
10	3.7
11	2.6
12	3.4
13	3.4
